# Effects of Pain-Reporting Education Program on Children's Pain Reports—Results From a Randomized Controlled Post-operative Pediatric Pain Trial

**DOI:** 10.3389/fped.2021.672324

**Published:** 2021-07-09

**Authors:** Dafna Zontag, Liat Honigman, Pora Kuperman, Roi Treister

**Affiliations:** ^1^Department of Nursing, Faculty of Social Welfare and Health Sciences, University of Haifa, Haifa, Israel; ^2^Department of Pediatric Surgery, Rambam Health Care Campus, Haifa, Israel

**Keywords:** pediatric, pain, pain measurement, pain scale, post-surgical

## Abstract

**Objective:** Accurate assessment of patients' pain is an essential part of adequate analgesic treatment. Although reporting pain is a complex task, limited-to-no instructions are provided to pediatric patients regarding this process. Our goal in this randomized parallel-group clinical trial (Clinicaltrial.gov study protocol number NCT04306679) was to evaluate if a training program designed to improve children's ability to understand and use pain scales in a post-surgical setting would affect their pain scores.

**Methods:** Eligible children (aged 8–17), hospitalized for elective surgery and their parents were randomized into two groups. Pre-surgery the intervention group underwent a multi-media program aimed to teach and train how to report pain. The control group received standard pre-surgical instructions. Post-surgery, the children reported their pain on 4 pain scales. The primary outcome was the concordance between children's pain intensity scores reported on four pain scales, both in terms of within-child standard deviation and absolute difference.

**Results:** Ninety-six children met inclusion criteria and completed the study. The trained subjects' pain reports had significantly (*p* = 0.002) lower within-subject standard deviation (0.41 ± 0.31) than the control group (0.67 ± 0.46). In line, regarding absolute difference, the concordance of children's pain reports was twice better in the trained group (mean difference of 0.43 ± 0.40) than in the control group (0.88 ± 0.70) (*p* < 0.001).

**Discussion:** Our results suggests that children's ability to report pain is a skill that can be improved. Future studies should test the potential clinical impacts of educational interventions aimed to improve pain assessment in children and adults.

## Introduction

Although evidence-based practice (1) surrounding pediatric perioperative care has continued to progress, improvements are still warranted (2, 3), with moderate to severe pain experienced by up to 40% of hospitalized children (4) and about 90 and 56% suffering some level of pain one- and 2-weeks following surgery (respectively) (5). This is concerning as there is evidence that early life pain and surgery can produce long-term changes both in sensory processing, as well as in future pain response (6, 7). The intensity of acute postoperative pain is one of the most recognized risk factors for the transition from acute to persistent post-surgical pain (PPSP) in both children and adults (8–14). Additionally, pediatric PPSP risk can be influenced by psychological [e.g., preoperative anxiety, negative emotional behaviors (1, 3, 9)], parent-related [e.g., higher pain catastrophizing by parents (1)] and patient-related [e.g., female sex, higher post-discharge pain (1, 3, 4, 8, 9)] factors. Analgesic interventions during the initial surgical period may therefore help prevent PPSP (1).

Appropriate and adequate post-operative pain treatment however relies on an accurate assessment of the patient's pain. With this, healthcare professionals have expressed impressions that patients variably understand and use pain scales, which is especially challenging given the vast normative cognitive development which transpires during childhood and adolescence (15). Regardless, it is important to allow children to play an active role in their own pain management. Previous work has found that children can adequately communicate regarding their desired postsurgical outcomes as well as pain level (2, 16, 17), and should be viewed as experts of their own pain management (18). In addition, proxy measures, recorded by a parent or medical staff, an often-used solution, present with inherent complexity. As such, like in adult pain management, self-reporting of pain remains the gold standard for children's pain measurement whenever feasible (19).

The use of a scale to rate one's pain is not an intuitive task, neither for adults, nor for children. It is often forgotten that the measurement tool for the assessment of pain intensity (and other subjective feelings) relies on the interaction between the subject using the scale, the scale itself and the context in which it is taken (20). For example, scores from the Numerical Rating Scale (NRS), the tool most frequently used among children aged eight and above (21–25), have been found to vary according to patient characteristics such as sex (26, 27) or ethnicity (28, 29), as well as the context (e.g., who is asking, parent or nurse) and their perception of, and experience with pain (30). A recent systematic review (21) concluded that while strong recommendations could be made for the use of the NRS for acute pain, only weak recommendations could be made regarding its use in postoperative and chronic pain. This may be due in part to the complex task which it entails, as the child must be able to both assess and transcribe the intensity of the pain into numbers (31) on a scale with a poorly defined upper limit.

Furthermore, when asking a patient to report their pain intensity, little-to-no instructions are commonly provided (32). Following this, our approach is that if an individual is properly educated (or “calibrated”), the reliability and validity of their pain intensity scores could be improved. Smith et al. were the first to test an intervention aimed at improving the quality of pain intensity scores. The efficacy of their intervention, tailored for implementation in clinical pain trials, was inconclusive (33). In a previous study by our group however, evoked-pain based training aimed to reduce the within-subjects variability of pain reports was found to be both effective and clinically relevant, as demonstrated in an adult cohort of painful diabetic neuropathy patients (34).

The aim of the current investigation was to assess if a pre-operational educational program focused on key elements of pain assessment could improve the concordance of children's post-operative pain intensity reports.

## Materials and Methods

### Patients

Children invited to participate in the study were hospitalized for elective surgery in one of the following four clinics: General pediatric surgery, Orthopedics, Ear-Nose-Throat (ENT), and Oral and Maxillofacial. Inclusion criteria: (1) children aged 8–17 years; (2) absence of psychiatric, cognitive, and/or neurological disorders; (3) ability to understand the purpose instructions of the study; (4) agree to participate and have at least one parent who agrees to sign informed consent. Based on power analyses (G^*^Power 3.1.9.2) for independent *t*-test model, with alpha of 0.05, power of 0.80, allocation ratio of 1 and medium effect size of 0.5, 102 subjects were needed. We chose to focus our intervention on children ages 8–17 because it has previously been suggested that due to cognitive development children under the age of eight may have a different understanding of pain scales and therefore introduce additional bias (15, 35).

### Intervention

The intervention was aimed to educate children on how to use the various pain scales and provide reliable pain assessments. It was based on a two short animation clips, lasting ~5 min each, and a short (5 min) guided interaction between the study nurse and the participants, in between the two clips. The intervention was available in the two major languages spoken in Israel (Arabic and Hebrew). Parents were present alongside their children during the intervention.

The first animation clip (http://clients.shivuknet.co.il/review/02b26f3c2f574a9625a7775058b200b6/version/1) focused on the importance of pain assessment, and introduce three pain scales [Categorical Pain Scale (CAT), Numerical Pain Scale (NPS) and Wong-Baker FACES Pain Rating Scale (FACES)], followed by explanations on their properties and the appropriate way to use them. Children were explained how to assign a number (or choose a category) that will best reflect their pain on each of the three scales. After the first movie, in order to reinforce what they had just learned about the use of pain scales, the study nurse performed a short exercise with the children. During this practice, the children were asked to recall three past painful events; corresponding to mild, moderate and severe pain and to report the magnitude of their pain during those events on the three pain scales. As the interventional program was provided to children of a range of ages, who were at varying developmental stages, the practice portion was tailored by the study nurse (DZ, a seasoned pediatric nurse) to the cognitive ability of each child. This was to reinforce that every child best understood the pain scales and how to use them, regardless of age. The second movie (http://clients.shivuknet.co.il/review/909e1e31ec208b28926bca57a97b0be5/version/1) aimed to provide an opportunity to implement their newly acquired knowledge. The child was requested to watch the clip and judge how well other children (in the movie) reported their pain.

In the control group, the children underwent the routine pre-operative preparations, which included a basic section on pain assessment in which the 0–10 numerical pain scale was introduced.

### Pain Assessment

Pain was reported by the children via pain diaries on four pain scales: (1) NPS (primary endpoint); (2) 0–100 mm Visual Analog Scale (VAS) (36); (3) FACES-Revised (37); and (4) a 5-point CAT (38) comprised of “no pain,” “mild pain,” “moderate pain,” “severe pain,” and “worst pain.” Children's were requested to report their pain in the surgery area. To allow comparison to the other scales, the responses on the CAT were transformed into a 0–10 scale by multipling the category (0, 1, 2, 3, 4) by 2.5. The data from the Wong-Baker FACES Scale was transformed into a 0–10 scale by multiplying each category (0–5) by 2. In line, the 0–100 VAS scale was divided by 10. We deliberately requested that the children report their pain on the VAS, which was not included in the educational training, to learn if training effects could be generalized to a scale not introduced in the program. For the same reason, the categorical scale used for data collection comprized five categories while in the training children's were trained on using a categorical scale with four categories. In addition, feedback about the training, and scale preference was collected prior to patient release.

During the study pain reports were also obtained from nurses and parents (see Clinicaltrial.gov study protocol number NCT04306679). Those results are not included in this manuscript as they are a topic of a future publication, focusing on differences in the assessment of pain between children, their parents, and nurses.

### Assessment of the Concordance of Children's Pain Scores Reported on Four Different Scales

Given the current lack of an objective gold standard for the assessment of pain, a direct assessment of how well one understands and uses pain scales to report their pain is not feasible. A close approximation can be obtained by assessing the concordance or discordance present in pain reports provided by the same patient at the same time on different pain scales (i.e., convergent validity) (24, 25).

Hence, we defined two proxy measures, representing the concordance of pain scores reported by each child on the four scales: (1) The within-subjects standard deviation (SD) between the four pain scores. To assure that variability is appropriately reflected by this measure, an additional sensitivity analysis excluded subjects who reported only “0” or “10” on all four scales due to potential bias caused by floor or ceiling effects; and (2) the average difference (calculated in absolute values) of the within-child pain reports. Specifically, the difference between the reported scores on each possible pairing of scales (NPS vs. VAS, NPS vs. CAT, NPS vs. FACES, VAS vs. CAT, VAS vs. FACES and CAT vs. FACES) was calculated in absolute values and averaged across the six pairs.

In addition to the two measures that focus on variability at the individual level, we also calculated the Pearson's correlation coefficients between each pairing for the entire cohort, and the trained and the untrained groups separately.

### Study Design

Experiments were conducted in accordance with the Declaration of Helsinki and with the approval of the Helsinki committee of Rambam HealthCare Campus (0091-19-RMB). Written informed consent was obtained from each participant before the beginning of this parallel study and an allocation ration of 1/1 was used for group assignment (intervention vs. control). Code numbers were allotted to protect patient annonymity.

All children, regardless of group, visited the outpatient clinic up to 3 days pre-surgery, as part of regular pre-operative preperations, which included basic instructions regarding pain assesment. At this opportunity, the children who were randomized onto the intervention group were exposed to the training program.

Post-surgery, 1 h following the administration of first analgesic (in response to first request of analgesic post-operation), the child recorded their pain level on the four pain scales which were laid out in a pain diary. In order to assure that each scale was indepentently completed, the scales were provided on separate pages, which were of adequate thickness to insure that the children could not discern their previous assesssment. The analgesics, provided as part of the clinical routine (not as a study drug) included Paracetamol, NSAID's and Opiates.

It should be noted that the study nurse was not blinded to the allocation of participants to the intervention or control arm, however, this is not likely to have introduced bias as she was not actively collecting the pain scores (they were independently recorded by the children). Before they were released from the hospital the children were also asked to provide feedback about the training on a 1–5 scale, with one representing “not satisfied at all” and 5 “very satisfied” as well as their preference of pain scale.

### Statistical Analyses

Data was processed and analyzed with SPSS for Windows version 23 (Chicago, IL). Descriptive statistics were used to present demographic and baseline characteristics. All measures were normally distributed (Shapiro-Wilk tests).

Independent-Samples *T*-tests were used to assess between-group differences in all dependent variables. Pearson's correlations were employed to assess the strength of correlations between each pain score pairing in the entire cohort, and separately in each group. Data is expressed as means ± SD or as percentages.

## Results

### Cohort Characteristics

**Figure 1:**
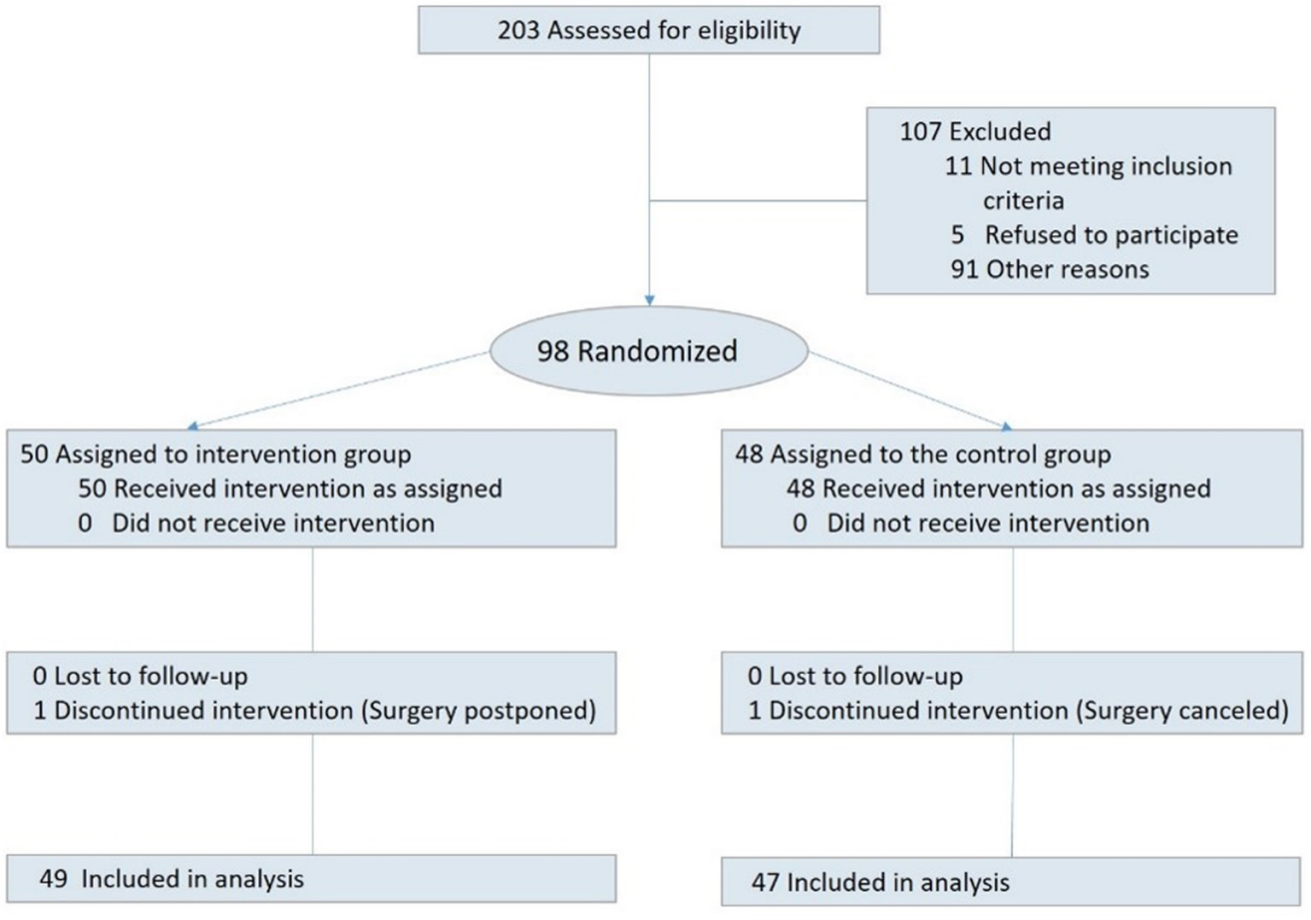
CONSORT study flow diagram.

Among 233 children who were admitted to the pediatric surgery departments between June and November 2019, 203 were eligible to participate. Among those, 81 children were not approached due to unavailability of the research assisant. The remaining 122 were invited to participate. Out of which, five refused, eight had their surgery canceled, and 11 were transferred to other units; consequently 98 children signed an informed consent. Two participants did not complete the study due to surgery cancelation or postponement. Hence 96 children successfully completed the study. Among those, 49 children were randomized into the intervention group, and 47 to the control group (for CONSORT study flow diagram see Figure 1).

Demographic and medical data of the 96 children, is summarized in Table 1. Approximately half of the cohort were native Hebrew speakers and the other half native Arabic speakers. The largest number of operations (44%) were in the Orthopedic department, about a quarter of the cases were in the general surgery (25%) and ENT (23%) pediatric departments. About half (49%) of the children underwent operations in the past. No differences were seen in any of the sociodemographic and medical characteristics between the control and intervention groups.

**Table 1:** Demographic and medical data.

**Characteristics**	**Total *n =* 96**	**Training *n =* 49**	**Control *n =* 47**	***P*-value**
Age (years)				0.560
Mean ± SD	13.15 ± 2.8	12.98 ± 2.8	13.32 ± 2.9	
Range	8–17	8–17	8–17	
Sex, *n* (%)				0.414
Male	46 (47.9%)	25 (54.4%)	21 (45.6%)	
Female	50 (52.1%)	24 (48.9%)	26 (55.3%)	
Native language, *n* (%)				0.849
Hebrew	50 (52.1%)	26 (53.0%)	24 (51.0%)	
Arabic	46 (47.9%)	23 (47%)	23 (49%)	
Former operation, *n* (%)				0.228
Yes	47 (48.9%)	24 (49.0%)	23 (49.0%)	
No	49 (51.0%)	25 (51.0%)	24 (51.0%)	
Department, *n* (%)				0.494
Orthopedic	42 (43.8%)	20 (41.0%)	22 (46.8%)	
General	24 (25.0%)	11 (22.4%)	13 (27.7%)	
ENT	22 (22.9%)	12 (24.4%)	10 (21.3%)	
OM	8 (8.3%)	6 (12.2%)	2 (4.2%)	

*ENT, ear-nose-throat, OM, oral and maxillofacial, SD, standard deviation. Data are n (%) or mean (SD); (n = 96)*.

### Children's Pain Reports on the Four Pain Scales

**Figure 2:**
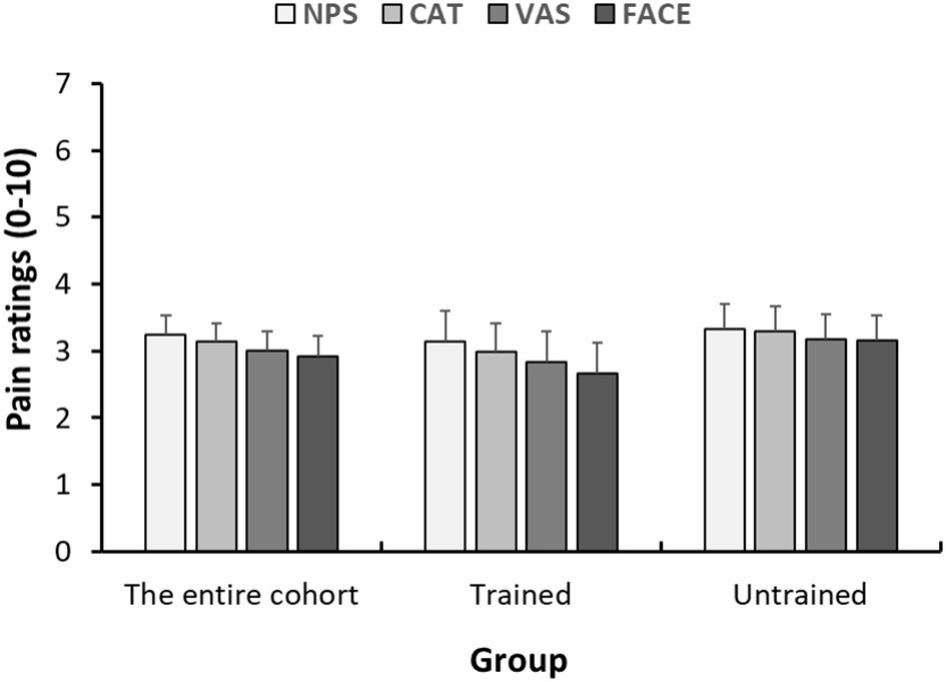
Within-child concordance of pain scores reported on various scales. Mean pain scores reported on the 4 pain scales (NPS, numerical pain scale; CAT, categorical scale; VAS, visual analog scale; FACES, face scale) 1 h after analgesic consumption. Each bar represents the average pain score. Error bars represent the standard error of the mean.

The means of the pain score reported on each of the four scales are presented in Figure 2. On average, children rated their pain intensity as 3.24 ± 2.60. No differences between the study groups were seen in pain intensity scores on any of the four scales (*P* > 0.05 for all). A summary of the means (±SD) of pain scores reported on each scale seperated by departments can be found in Supplemantary Table 1.

### Within-Child Concordance of Pain Intensity Scores

#### Within-Child SD

The average within-subjects' standard deviation in the entire cohort was 0.53 ± 0.41, with a significant between-groups difference (*p* = 0.002). The trained subjects' pain reports had a significantly lower within-subject standard deviation (0.41 ± 0.31) than the control group (0.67 ± 0.46). As a sensitivity analysis, we then completed a second analysis where we excluded children that reported 0 or 10 on all four scales, since in those individuals, the concordance is biased due to a ceiling or floor effect. Among the 68 children who entered this analysis (*n* = 32 in the trained and *n* = 36 in the untrained groups), the average within-subjects' standard deviation in the entire cohort was 0.69 ± 0.33. In this sensitivity analysis as well, significant between-groups difference was found (*p* < 0.001). The trained subjects' pain reports had a significantly lower standard deviation (0.55 ± 0.22) than the control group (0.82 ± 0.36).

#### Within-Child Average Difference Between Pain Reports

**Figure 3:**
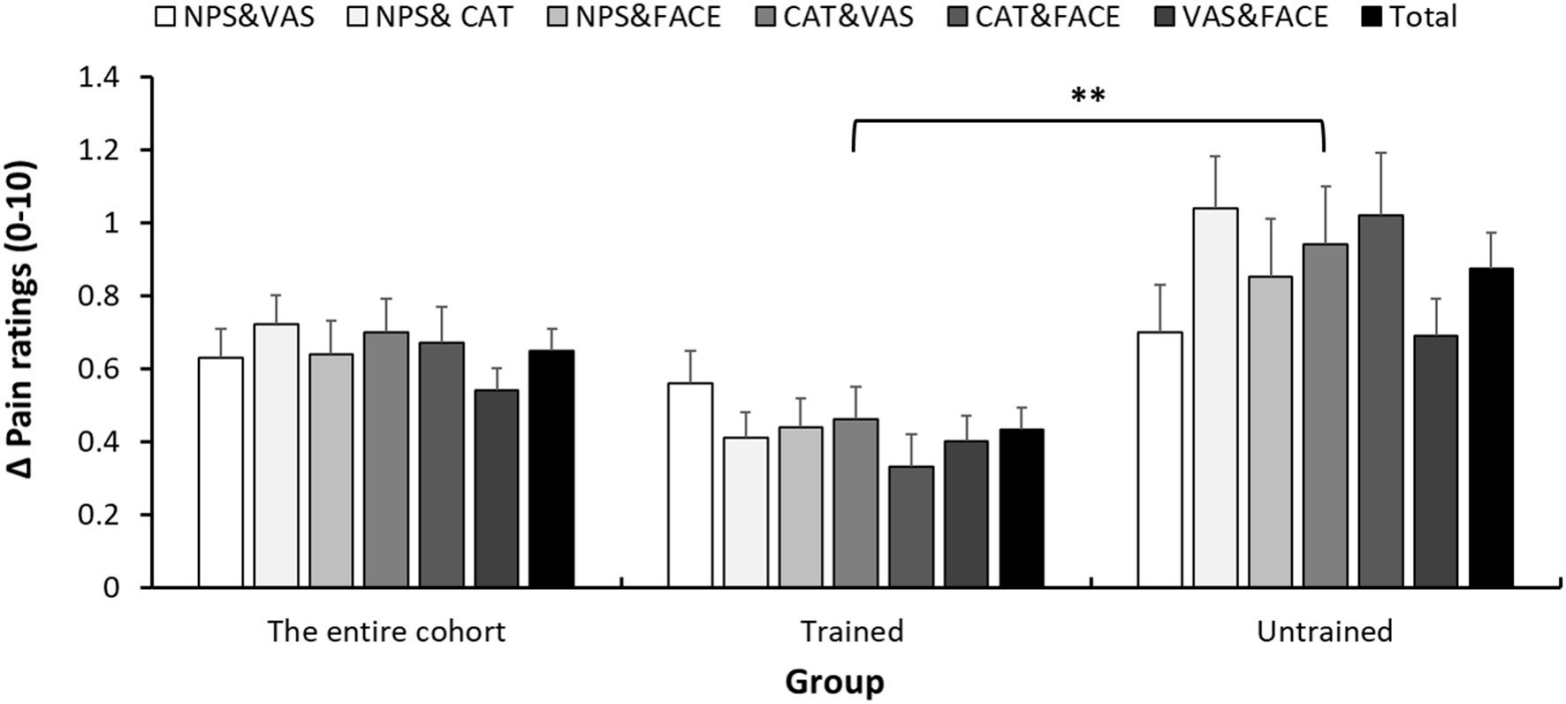
Within-child average difference between pain reports. Differences in pain scores between each pair of pain scales (NPS, numerical pain scale; CAT, categorical scale; VAS, visual analog scale; FACES, face scale), 1 h after analgesic consumption. Error bars represent the standard error of the mean. **P < 0.01.

Figure 3 presents the within-child differences in pain intensity reports. Those differences were calculated as the difference between pain scores reported on two scales, in absolute values. In the entire cohort, the mean difference was 0.65 ± 0.60. Those differences were significantly (*p* < 0.001) lower in the trained (0.43 ± 0.40) than in the untrained (0.88 ± 0.70) group. Given that average pain intensity was about 3.2 (on the 0–10 scale), in terms of percentage of change in pain, those differences are in the range of 13 and 28% in the trained and untrained, respectively. A summary of the within-child differences in pain intensity reports between each of the pairs of pain scales seperated by departments can be found in Supplemantary Table 2. In addition, we were interested to explore if, among the trained group, the training benefits could be extrapolated onto the VAS, which was not covered by the training. As can be seen in Figure 3, differences between each pair of scales, regardless if the VAS is included or not, were lower in the trained group. In addition, in the trained group, we compared the average difference (in absolute value) of the three pairings of pain scores that included the VAS (VAS vs. FACES, VAS vs. CAT and VAS vs. NPS) to the average of the three pairings which do not include the VAS (NPS vs. FACES, NPS vs. CAT and FACES vs. CAT). No significant differences were found between these averages (0.48 ± 0.48 in the pairing including the VAS and 0.39 ± 0.45 in the pairing not including the VAS, *p* = 0.120).

#### Group-Level Coefficient Correlations Between the Four Pain Scores

The coefficient correlations between each pair of pain scores reported on the different scales (NPS vs. VAS, NPS vs. CAT, NPS vs. FACES, VAS vs. CAT, VAS vs. FACES and CAT vs. FACES) are presented in Table 2. In the entire cohort, the coefficient correlations were in the range of 0.862–0.934 for all six pairings. In the trained group, the correlation coefficients were in the range of 0.928–0.970 while in the untrained group the same pairing produced correlations in the range of 0.728–0.903.

**Table 2:** Group-level coefficient correlation between the pain reports.

	**NSP**	**Cat**	**Faces**	**VAS**
**In the entire cohort**				
NSP	1	0.889^**^	0.892^**^	0.918^**^
CAT		1	0.862^**^	0.862^**^
FACES			1	0.934^**^
VAS				1
**In the trained group**				
NSP	1	0.963^**^	0.970^**^	0.954^**^
CAT		1	0.946^**^	0.928^**^
FACES			1	0.961^**^
VAS				1
**In the untrained group**				
NSP	1	0.774^**^	0.800^**^	0.876^**^
CAT		1	0.728^**^	0.764^**^
FACES			1	0.903^**^
VAS				1

** *p < 0.001*.

#### Children's Preferences of Pain Scale

Most of the children (55.1%) preferred the NPS scale; nearly a quarter of the children (22.4%) had no specific preference, while about 10% of the children preferred either the CAT (12.2%) or the FACES (8.2%). Only fraction of the children (2.0%) preferred the VAS. To investigate if the developmental stage related to the children's preference of a pain scale, we divided the cohort into younger vs. older children (splitting the cohort by median = 14 years) and found no relationship between age and scale preference (results not shown).

#### Children Satisfaction With the Instructions Provided Regarding Pain Assessment

**Figure 4:**
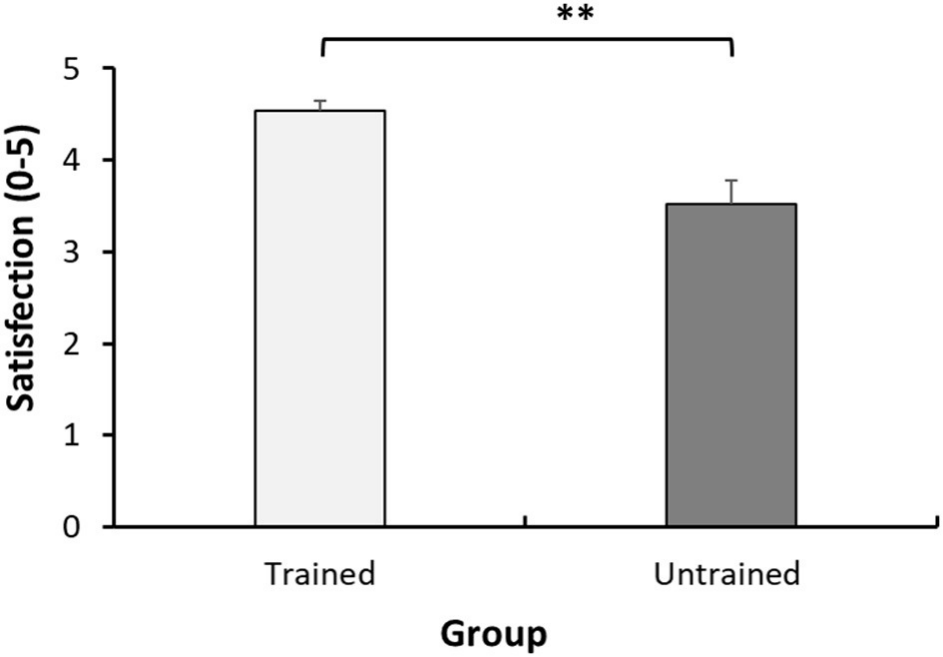
Satisfaction from pre-operative pain instructions. Mean satisfaction scores (0–5) of the child in each group. Each bar represents the mean. Error bars represent the standard error of the mean. **P < 0.01.

A significant difference was found in the general satisfaction (rated 0–5) from the pre-operative pain guidance children received, between the trained and untrained group (4.54 ± 0.60 vs. 3.52 ± 1.40, *p* = 0.004) (see Figure 4). At the end of the study, the children were requested to provide qualitative feedback on the training as well. Overall, the children were satisfied, and perceived the training as helpful in describing their pain, innovative and engaging.

## Discussion

This report summarizes the results of the first pediatric study aimed at improving children's understanding of the use of pain scales to report their pain intensity. Our underlying assumption was that the measurement tool for the assessment of pain is not only the scale used—rather, it is the interaction between the scale, the one using it, and the context (20). While it is intuitive that measurement tools require periodic calibration, to date there have been virtually no attempts to “calibrate” the patients using pain scales, although they are an integral part of the pain measurement tool.

The results from this study support our underlying assumption: The pre-operational educational training was found to be effective in improving the concordance of post-operative pain reports. Improved concordance implies that the training improved the children's ability to report their pain, regardless of the scale used. To date there seems to be no clinical tool in use which assesses a child's cognitive ability to understand and use pain scales which are integral for their hospitalization. We are also unaware of other pediatric studies in which an intervention that was aimed to improve symptoms reporting was assessed. There is one study in an adult population (33), in which chronic pain patients were trained to better report their pain, in the context of clinical trials, but the efficacy of the intervention was questionable.

Given the lack of objective gold standard to compare with, we rely on the concordance between reports provided on different scales to assess the reliability of pain reports. While the 4 pain scales used in the current study might capture slightly different aspects of pain intensity (e.g., the FACES scale might include more affective components), it is reasonable to use this approach to assess the reliability of pain reports (24, 25). For example, in a study validating the NPS in a pediatric post-operative setting Pagé et al. relied on convergent validity between the NPS, VAS, and faces resting scales, and absolute pain score difference between NPS and FACES as part of their verification process (25). A later study by Tsze et al. further explored the validity and reliability of the NPS in children with acute pain, relying as well on convergent validity between NPS and FACES and the simple agreement between the pain scores (24). Alongside other measures, such as the total proportion of incorrectly ranked ratings (i.e., ratings which appeared in the appropriate order of least ≤ average ≤ worst) Smith et al. assessed convergent validity as well as a marker for the outcome of their interventional trial (33). As such, it could be concluded that among the various potential strategies to assess the reliability of pain scores, none are perfect and studies often employ several variations in order to assure rigor.

We used two measures to assess concordance between pain reports, reflecting slightly different aspects and holding different limitations and advantages. When the concordance is assessed in terms of absolute difference between reports provided on the different scales, the interpretation is clear, since it is given in terms of 0–10 pain scores. In this study, the difference between pain reports reported on different scales was twice larger (28%) in the control group than in the trained group (13%). While the clinical implications are yet to be determined, in terms of research, reduced within-subject SD improves statistical power and reduce the number of subjects and research needed for a study.

During the training children were exposed to instructions on how to use three pain scales (NPS, CAT, and FACES scales), but during the study they were requested to report their pain on four scales (including the VAS). This was done with the aim of exploring whether training effects could be extended to the use of pain scales not covered by the training. Notably, we found no difference in the concordance between the pain scores, which were covered by the training program, and the concordance of the VAS with all other scales. This suggests that the implementation of a training program which teaches and trains children regarding the use of pain scales may have applications which exceed the specific content of the program, as pain reporting is a skill which can be honed.

We are unaware of other studies aimed at improving children's use of pain scales to report their pain. A few studies however, have assessed the effects of other educational programs in this population. Several of which, such as those centered around the provision of pre-surgical information sheets, focused on the parent's role in the care process, were found not to influence children's pain levels or functional outcomes (39–42). Others (43, 44) were child-centered, but focused around general pain education or transcription of children's pain experiences. For example, Crandall et al. examined the effects of a pre-operative pain education booklet on the children's self-reported post-operative clinical outcomes including pain expectation. While the majority of the children in the intervention reported that the pre-operative education helped with their post-operative pain, no significant differences between the groups was observed for pain intensity (43). Our work, by contrast, is innovative both in the design, which provides training on how to report pain, and the results, which saw a significant difference between the intervention and control groups.

In terms of preference, among the unidimensional pain scales that were used in this study, children preferred the Numerical Pain Score over other scales. This is in line with literature supporting its validity in pediatric pain assessment (21). In addition, the children who received training were more satisfied with the pre-surgical instructions than their peers who received the routine pre-operation guidance. This satisfaction serves as preliminary evidence that an intervention such as the one presented here, would not only be helpful for, but also well-tolerated by, pediatric populations.

There are limitations, which deserve consideration: First, the study nurse who administrated the training and collected the data (DZ) was not blinded to the children's allocation to training/control groups. However, the children were requested to independently report their pain, via pain diary, with no involvement of the study nurse, so it is unlikely that her knowledge influenced their reporting. Furthermore, the children were unaware that the main study outcome was the concordance between pain scores, so their reports can be assumed to be free of bias. Second, the study is not powered, given the limited number of participants in some departments, to compare the results between departments or kind of surgery. Lastly, the sample size may not be sufficient to support the statistical conclusions.

It is often forgotten that reporting of subjective feelings, such as pain, on a unidimensional 0–10 scale is a complex task: one needs to direct attention inwardly toward the body, focus on the feeling being assessed (e.g., pain, mood) and assign it a single number on a scale that is variably understood. The current study suggests that the assessment of pain, and probably other subjective symptoms, is a skill that should be nurtured. While the clinical effects of such intervention are yet to be determined, attention to the patients understanding and use the provided scales should improve the assessment, diagnosis and outcomes of patients suffering from symptoms that could not be objectively measured.

## Data Availability Statement

The data of this study will be available from the corresponding author, upon reasonable request.

## Ethics Statement

The studies involving human participants were reviewed and approved by Helsinki committee of Rambam HealthCare Campus. Written informed consent to participate in this study was provided by the participants' legal guardian/next of kin.

## Author Contributions

DZ: study design, patient recruitment, and writing and review of manuscript. LH: study design, data analysis, and review of manuscript. PK: data analysis and writing of manuscript. RT: study design, study oversight, data analysis, and writing and review of manuscript. All authors contributed to the article and approved the submitted version.

## Conflict of Interest

The authors declare that the research was conducted in the absence of any commercial or financial relationships that could be construed as a potential conflict of interest.
