# Supplementary material for: Effects of Pain-Reporting Education Program on Children's Pain Reports—Results From a Randomized Controlled Post-operative Pediatric Pain Trial
Source: Front Pediatr. 2021 Jul 9;9:672324. doi: 10.3389/fped.2021.672324 (PMC8298899; doi:10.3389/fped.2021.672324)
Supplement: Supplementary file 1 [file Table_1.DOCX]

**Supplementary table S1**

**Mean pain scores reported in the entire cohort, the trained and untrained groups, by department**

| **Entire cohort (n=96)** | | **Orthopedic (n=42)** | **General (n=24)** | **ENT (n=22)** | **OM (n=8)** |
| --- | --- | --- | --- | --- | --- |
|  | **NPS** | 3.61 (2.48) | 2.67 (2.61) | 3.17 (2.46) | 3.40 (3.58) |
|  | **CAT** | 3.26 (1.97) | 2.93 (2.68) | 3.19 (2.24) | 3.00 (3.26) |
|  | **Faces** | 3.42 (2.41) | 2.43 (2.48) | 3.42(1.95) | 3.20 (3.63) |
|  | **VAS** | 3.47 (2.63) | 2.35 (2.43) | 2.77 (2.50) | 3.39 (3.60) |
| **The trained group (n=49)** | | **n=21** | **n=11** | **n=12** | **n=6** |
|  | **NPS** | 3.12 (0.37) | 2.91 (3.39) | 3.00 (2.79) | 4.25 (3.50) |
|  | **CAT** | 3.09 (2.08) | 2.50 (3.12) | 3.00 (2.58) | 3.75 (3.23) |
|  | **Faces** | 2.71 (2.11) | 2.20 (3.05) | 2.54 (2.38) | 4.00 (0.36) |
|  | **VAS** | 3.15 (2.61) | 2.36 (3.14) | 2.39 (2.91) | 2.87 (2.71) |
| **The untrained group (n=47)** | | **n=21** | **n=13** | **n=10** | **n=2** |
|  | **NPS** | 4.00 (2.55) | 2.46 (1.85) | 3.37 (2.13) | 0.00 (-) |
|  | **CAT** | 3.42 (1.90) | 3.27 (2.37) | 3.44 (1.86) | 0.00 (-) |
|  | **Faces** | 4.00 (2.53) | 2.61 (2.06) | 2.25 (1.28) | 0.00 (-) |
|  | **VAS** | 3.74 (2.68) | 2.34 (1.77) | 2.72 (1.79) | 4.15 (5.88) |

ENT, ear-nose-throat, OM, oral and maxillofacial; values are means, standard deviation values presented in parentheses. Analysis of Variance (ANOVA) test revealed no differences between the departments in any of the outcomes.
