# Supplementary material for: Effects of Pain-Reporting Education Program on Children's Pain Reports—Results From a Randomized Controlled Post-operative Pediatric Pain Trial
Source: Front Pediatr. 2021 Jul 9;9:672324. doi: 10.3389/fped.2021.672324 (PMC8298899; doi:10.3389/fped.2021.672324)
Supplement: Supplementary file 2 [file Table_2.DOCX]

**Supplementary table S2**

**Mean differences between pain scores reported on each two scales in absolute values in the entire cohort, the trained and untrained groups, by department**

| **Entire cohort** |  | **Orthopedic (n=42)** | **General (n=24)** | **ENT (n=22)** | **OM (n=8)** |
| --- | --- | --- | --- | --- | --- |
|  | **NPS & VAS** | 0.62 (0.80) | 0.80 (0.73) | 0.67 (0.78) | 0.10 (0.21) |
|  | **NPS & CAT** | 0.73 (0.81) | 0.85 (0.91) | 0.70 (0.77) | 0.31 (0.46) |
|  | **NPS & Face** | 0.57 (0.66) | 0.75 (0.99) | 0.86 (1.21) | 0.12 (0.35) |
|  | **CAT & VAS** | 0.74 (0.93) | 0.70 ( 0.97) | 0.78 (1.02) | 0.20 (0.43) |
|  | **CAT & Face** | 0.64 (0.86) | 0.77 (1.14) | 0.80 (1.18) | 0.19 (0.37) |
|  | **VAS & Face** | 0.54 (0.48) | 0.46 (0.52) | 0.75 (0.87) | 0.22 (0.45) |
|  |  |  |  |  |  |
| **The trained group** |  | **n=21** | **n=11** | **n=12** | **n=6** |
|  | **NPS & VAS** | 0.49 (0.48) | 0.79 (0.76) | 0.69 (0.74) | 0.13 (0.24) |
|  | **NPS & CAT** | 0.40 (0.50) | 0.45 (0.52) | 0.37 (0.48) | 0.42 (0.50) |
|  | **NPS & Face** | 0.52 (0.51) | 0.36 (0.50) | 0.50 (0.67) | 0.17 (0.41) |
|  | **CAT & VAS** | 0.45 (0.53) | 0.36 (0.55) | 0.66 (0.97) | 0.27 (0.48) |
|  | **CAT & Face** | 0.40 (0.66) | 0.10 (0.30) | 0.46 (0.89) | 0.25 (0.42) |
|  | **VAS & Face** | 0.39 (0.43) | 0.30 (0.32) | 0.58 (0.64) | 0.30 (0.50) |
|  |  |  |  |  |  |
| **The untrained group** |  | **n=21** | **n=13** | **n=10** | **n=2** |
|  | **NPS & VAS** | 0.73 (1.00) | 0.81 (0.74) | 0.64 (0.85) | 0.00 (-) |
|  | **NPS & CAT** | 1.02 (0.93) | 1.20 (1.03) | 1.10 (0.87) | 0.00 (-) |
|  | **NPS & Face** | 0.60 (0.78) | 1.08 (1.19) | 1.30 (1.57) | 0.00 (-) |
|  | **CAT & VAS** | 1.01 (1.13) | 0.10 (1.17) | 0.92 (1.12) | 0.00 (-) |
|  | **CAT & Face** | 0.85 (0.97) | 1.35 (1.28) | 1.20 (1.40) | 0.00 (-) |
|  | **VAS & Face** | 0.68 (0.48) | 0.61 (0.61) | 0.96 (1.08) | 0.00 (-) |
|  |  |  |  |  |  |

ENT, ear-nose-throat, OM, oral and maxillofacial; values are means, standard deviation values presented in parentheses. Analysis of Variance (ANOVA) test revealed no differences between the departments in any of the outcomes.
